# Psychometric Properties of a Multidimensional Scale of Sense of Community in the School

**DOI:** 10.3389/fpsyg.2017.01466

**Published:** 2017-08-29

**Authors:** Gabriele Prati, Elvira Cicognani, Cinzia Albanesi

**Affiliations:** Department of Psychology, University of Bologna Cesena, Italy

**Keywords:** school sense of community, factor analysis, reliability, school, well-being

## Abstract

Sense of community in the school has been associated with a range of important outcomes for students. However, there are currently no standard definitions of sense of community in the school with corresponding measures with established psychometric properties. To fill this gap, the main aim of the present study was to propose a model of sense of community in the school, its operationalization and to examine its key psychometric properties (factorial structure, reliability, differential item functioning, differential test functioning of the scale and discriminant, convergent, and criterion validity). Participants were 1,076 students from 22 public middle schools and 724 students from 22 public high schools located in the Italian city of Genoa and its province. To test the dimensionality of the scale, we conducted exploratory and confirmatory factor analysis under the Item Response Theory paradigm. Exploratory factor analysis confirmed three dimensions: Membership, Emotional connection, and Opportunities. A confirmatory factor analysis revealed that the bifactor model exhibited the largest improvement in fit. Cronbach’s alpha, omega total, and omega hierarchical indicated a good reliability for the measure. Internal consistency was satisfactory, considering Cronbach’s alpha and omega. Analysis of differential item/test functioning of the scale showed that girls and boys as well as students attending middle school and those attending high school responded in largely similar ways to the measure. Finally, the instrument demonstrated good discriminant, convergent, and criterion validity. Together, these findings indicate that our theory driven model of sense of community in the school is valid and that the instrument is a reliable measure for assessing sense of community in the school.

## Introduction

Many researchers have used the concept of sense of community to describe the psychological aspects of physical and social contexts that satisfy the need of belonging (Fisher et al., 2002). Sense of community was defined by McMillan and Chavis (1986) as “the feeling that members have of belonging, a feeling that members matter to one another and to the group, and a shared faith that members’ needs will be met through their commitment to be together” (p. 9). This definition in principle can apply to school, that can be considered as a primary physical and social context for young people, and can be adapted as follows: it refers to feeling of belonging to the school as a community, perception of emotional connection and bonds with other students, and the feeling that personal needs are satisfied through such membership. Concerning membership, McMillan and Chavis (1986) argue that “membership has boundaries; this means that there are people who belong and people who do not. The boundaries provide members with the emotional safety necessary for needs and feelings to be exposed and for intimacy to develop.”

Sense of community in the school has been related to a range of important outcomes for students (Baker, 1998; Osterman, 2000; Bateman, 2002). For instance, previous investigations revealed that psychological sense of community in the school is associated with democratic school climate (Vieno et al., 2005), lower levels of drug use and delinquent behavior (Battistich and Hom, 1997), lower levels of bullying behaviors (Vieno et al., 2013; Wang et al., 2014), academic achievement (Battistich et al., 1995; Wang et al., 2014), engagement in school activities and role clarity (Royal and Rossi, 1996), social support (Pretty et al., 1996; Vieno et al., 2007), quality of peer interactions (Hallinan et al., 2008), social skills (Battistich et al., 1995), lower levels of loneliness (Pretty et al., 1994, 1996), self-efficacy (Vieno et al., 2007), and well-being (Pretty et al., 1996; Royal and Rossi, 1996; Vieno et al., 2007, 2013).

According to McMillan and Chavis (1986), sense of community is a multidimensional construct, which includes four dimensions like sense of belonging (membership), shared emotional connection with the community, perceived influence over it, and perceived opportunities for satisfying one’s needs through such belonging (integration and fulfillment of needs). Research conducted on residential sense of community among adolescents (Albanesi et al., 2007; Chiessi et al., 2010), using the sense of community scale for adolescents confirmed the multidimensionality of the construct.

Despite these conceptual and methodological efforts, as researchers have sought to investigate sense of community in the school, each study used a unique measure of this construct which was usually operationalized as a single-factor construct. The development of the scale was not usually theory driven but based on empirical findings (see Hagborg, 1998; Rovai et al., 2004). In addition, other studies used typically *ad hoc* scales devised by researchers for a specific study, and few data are available on their validity. For instance, Pretty et al. (1994, 1996) used a single factor score in their school-referenced version of sense of community. Recently, a self-report measure of classroom sense of community for adolescents has been validated (Petrillo et al., 2016). However, classroom sense of community and sense of community in the school are two distinct constructs, as the former does not capture the overall perception of the quality of the school environment (Hagborg, 1998). Specifically, classroom sense of community mainly assessed the relationships among students and with teachers and classroom climate. Sense of community in the school focuses on sense of belonging to the school as a community, emotional connection and bonds with other students of the school, and the perception that personal needs are satisfied through such membership.

Given that the theory proposes that sense of community is multidimensional (McMillan and Chavis, 1986; Fisher et al., 2002; Tartaglia, 2006), our first aim was to propose a conceptualization and an associated measure of sense of community in the school including three dimensions: membership (feeling of belonging to and identification with the school, perception of emotional safety), emotional connection (the relationships and emotional bonds established with other members of the school community), and opportunities (the perception of enjoying opportunities for satisfying needs and have influence through such membership). To this end, we aim at investigating factorial structure and reliability of a theory driven scale of sense of community in the school. Exploratory and confirmatory factor analytic techniques are commonly used to determine the factorial structure of an instrument. In the present study, we will also use bifactor and item response theory analyses (Gibbons et al., 2007; Immekus and Imbrie, 2008; Reise, 2012) to determine the extent to which item responses on a scale of sense of community in the school are saturated by a general factor, and the degree to which multidimensionality is meaningful. In a bifactor structure, each item loads on a general factor that, therefore, accounts for the commonality shared by the group factors, as well as on one of the two or more orthogonal group factors, each of which accounts for the unique effect of the group factor after partialling out the general factor. According to Chen et al. (2006), bifactor models are particularly well suited to testing multidimensional constructs. A bifactor model allows researchers to identify whether group a specific factor remains a unique contributor, after taking into account the common variance shared with other group factors, and, therefore, is useful to empirically examine the opportunity of forming subscales (Reise et al., 2007; Chen et al., 2012; Reise, 2012). A bifactor model can provide evidence for the co-existence of the general factor and the relatively distinct nature of each group factor.

Differences according to gender and age group in the scores of sense of community have most typically been investigated (Baker, 1998; Osterman, 2000; Bateman, 2002). Different scholars (e.g., Pretty et al., 1996; Chipuer and Pretty, 1999; Hughey et al., 1999; Sánchez et al., 2005; Albanesi et al., 2007; Peterson et al., 2008; Chiessi et al., 2010) have raised questions about the stability of the theoretical dimensions across contexts and with different groups (e.g., gender, age). One the one hand, these differences according to gender and age group in the scores of sense of community may be due to socio-psychological processes, for instance high school students may be less interested in fitting into the school environment than middle school students (Sánchez et al., 2005). On the other hand, it is not clear to what extent the content of an item affects the item endorsement of sub-groups of adolescents based on gender or age group. Differential item/scale functioning refers to the extent to which the item/scale might be measuring different aspects for members of separate subgroups. Previous studies investigating sense of community among adolescents did not examine differential item/scale functioning. Therefore, the second aim involves determining whether the items and the scale of sense of community in the school are measuring in essentially the same way for all subgroups. To this end, using an item response theory approach, we assessed differential item functioning (DIF) and differential test functioning (DTF). DIF refers to any situation in which an item within a scale performs differently for members of different groups who have the same level of the latent trait of the construct. This logic is extended to DTF where the invariance of the performance of a set of items (total score) is evaluated across different groups.

Finally, a third aim of the study is to examine discriminant, convergent, and criterion validity. This aim involves empirically examining the degree to which sense of community in the school diverges from other related constructs such as satisfaction with the classroom environment and students’ relationships with their teachers. Thus, to investigate discriminant validity, we will use the scale of satisfaction with the classroom environment and the scale of students’ relationships with their teachers (Cicognani and Albanesi, 2015). Since evidence of convergent validity is best interpreted relative to discriminant evidence (John and Benet-Martínez, 2000), the patterns of intercorrelations between the dimensions of the scale of sense of community in the school should be greater while correlations with the scale of satisfaction with the classroom environment and the scale of students’ relationships with their teachers (Cicognani and Albanesi, 2015) should be substantially lower. In terms of criterion validity of the instrument, research has reported associations between sense of community referred to the residential community and well-being (Davidson and Cotter, 1991; Pretty et al., 1996; Prezza and Costantini, 1998; Prezza et al., 2001; Chipuer et al., 2003; Farrell et al., 2004; Albanesi et al., 2007; Cicognani et al., 2009; Prati et al., 2015). In addition, there are also theoretical reasons to expect that well-being is an outcome of interest for sense of community in the school. Jorgensen et al. (2010) proposed a model of well-being which shows that sense of community is the most proximal variable to well-being. In addition, according to the conceptual framework proposed by Vieno et al. (2007), sense of community in the school may predict well-being. Since criterion validity refers to the ability of a measure to predict the outcome of interest, in the present study we focus on the relationship between sense of community in the school and well-being. Specifically, to investigate the degree to which the construct of sense of community in the school is associated with well-being (criterion validity), we will use the Mental Health Continuum–Short Form (Keyes, 2006; Keyes et al., 2008; Lamers et al., 2011) as the measure of well-being.

## Materials and Methods

### Participants

The sample for this study includes 1,076 students from 22 public middle schools (Grade 2, corresponding to 12–13 years of age) and 724 students from 22 public high schools (Grade 4, corresponding to 17–18 years of age) located in the Italian city of Genoa and its province. Male students were 998 (55.4%), while female students were 802 (44.6%). A clear majority of students (*n* = 1,673; 92.9%) was born in Italy.

### Measures

The following were the measures included in the questionnaire.

The Scale of Sense of Community in the School (SoC-S) was developed to measure students’ sense of the school as a community as defined in the introduction. Supplementary Material provides the full item set. To assess the three dimensions of sense of community in the school, we adapted part of the items of the scale of Sense of Community on adolescents (Cicognani et al., 2006; Albanesi et al., 2007; Chiessi et al., 2010) that measure the dimensions of membership, emotional connection, and opportunities. Specifically, 10 items were selected for their theoretical relevance and adapted to assess sense of community in the school (instead of residential sense of community). We considered the attributes of the construct’s dimension that are more relevant for the school community and seem to better capture the psychological needs that fit the school mission/environment. We included items measuring emotional safety (“I feel safe in my school”), sense of identification (“I am proud to belong to this school”) and a measure of the extent to which students share positive representations (“I think this is a good school”), thus contributing to a commons symbolic system. These three attributes contribute to membership (McMillan and Chavis, 1986). Other items measured the attributes of emotional connection: we included a measure of contact (“I spend a lot of time with other students at school”), a measure of quality of interaction (“I like to stay with other students attending this school”), and a measure of spiritual bond (“In this school, I feel I can share experiences and interests with other students”). Then we measured opportunities of integration and fulfillment of needs: based on the idea that fulfillment of needs acts as a reinforcement of sense of community, we identified four items that refer to different psychological needs in adolescence (to be listened to, to have positive experiences, to explore between different options). The item “Students are involved in organizing a variety of school events” refers to the need to participate and have an active role. Therefore, we decided to use four items because the dimension of Opportunity is complex. Responses were rated on a 5-point scale (1 = *not at all true*, 2 = *slightly true*, 3 = *fairly true*, 4 = *very true*, 5 = *completely true*).

Students’ relationships with their teachers were measured using a 3-item scale derived from previous research (Cicognani and Albanesi, 2015). The items are the following: “My teachers are fair and just,” “My teachers are interested in me as a person,” and “I have a good relationship with teachers.” In the present study, Cronbach’s alpha coefficient for this scale was 0.81.

Satisfaction with the classroom environment was assessed using a 4-item scale derived from previous research (Cicognani and Albanesi, 2015). The items that make up the scale are the following: “In my classroom, I feel good,” “In my classroom, I feel like a stranger,” “The students in this class really care about each other,” and “The students in my class enjoy being together.” In the present study, the alpha coefficient for this scale was 0.73.

Well-being was measured using the Mental Health Continuum–Short Form (Keyes, 2006; Keyes et al., 2008; Lamers et al., 2011). The instrument assesses positive mental health and, in the present study, showed good reliability coefficients (α = 0.86). Respondents rate the frequency of every feeling in the past month on a 6-point Likert scale from 1 (never) to 6 (always).

### Procedure

The ethics committee of the University of Bologna approved the study with written informed consent from all participants. All participants gave written informed consent in accordance with the Declaration of Helsinki. The school headmasters of middle and high schools of the city of Genoa and its province were contacted to ask permission to conduct the study. The school headmasters of 22 public middle schools and 22 public high schools gave their permission. Survey proctors assisted students, answered any questions about the questionnaire, and ensured confidentiality of responses. Before answering the questionnaire, students were told that the aim of the study survey was to gain information about their perception of their life in the school. Students were also assured about the confidentiality of their responses.

### Statistical Analyses

Analyses were conducted using R (R Core Team, 2016) and the packages *psych* (Revelle, 2013), *mirt* (Chalmers, 2012), and *psy* (Falissard, 2015). The sample was randomly split into two samples of approximately equal size: a “training sample” (*N* = 871) and a “holdout sample” (*N* = 929). We fit an unconditional maximum likelihood exploratory factor analysis model under the item response theory paradigm followed by oblimin rotation on the training sample. The log-likelihood test and four information criteria were used for the identification of the number of factors to be extracted in exploratory factor analysis and for model comparisons in subsequent confirmatory factor analysis. The four information criteria were as follow: (a) the Akaike information criterion (AIC), (b) the corrected AIC (AICc), (c) the Bayesian information criterion (BIC), and (d) the Sample-Size Adjusted BIC (SABIC). Lower values of the information indices indicate better fit. We performed confirmatory analysis for the holdout sample to confirm the model identified from the exploratory factor analysis. Confirmatory factor analysis was conducted using a multidimensional latent trait model under the item response theory paradigm. To evaluate model fit in applications of confirmatory IRT model when the items are polytomous, we used the M_2_^∗^ test statistic (Cai and Hansen, 2013) as well as associated fit indices RMSEA, CFI, and TLI that are based on M_2_^∗^. A bifactor model was included in the confirmatory factor analysis. Finally, we examined sex-based and age group-based (i.e., middle versus high school) DIF. To identify items with significant DIF, we used the Wald approaches with Benjamini–Hochberg adjustment. To evaluate overall bias in the SoC-S, we calculated the signed DTF (sDTF) and unsigned DTF (uDTF). sDTF is a measure of test scoring directional bias (i.e., negative values of sDTF indicate that the scores are biased in favor of one group, while positive values indicate that the scores are biased in favor of the other group), while the uDTF is a measure of the average absolute bias, irrespective of which group it favors. The statistical significance of sDTF was evaluated. The reliability was investigated using Cronbach’s alpha and omega (Zinbarg et al., 2005; Revelle and Zinbarg, 2009). Omega is an index of internal consistency such as Cronbach’s alpha. However, compared to Cronbach’s alpha, omega makes fewer and more realistic assumptions (e.g., the assumptions of the essentially tau-equivalent model) and has less risk of overestimation or underestimation of reliability (Raykov, 1997; Zinbarg et al., 2005; Revelle and Zinbarg, 2009). Thus, to consider how well a test measures one concept, it is recommended (e.g., Dunn et al., 2014) the use of McDonald’s omega total (ω_t_), an estimate of total reliability, because it permits the assessment of the extent to which composite scale scores are interpretable as a measure of a single common factor. In addition, when item response data are consistent with a bifactor structure, we calculated omega hierarchical (ω_h_) because it is an estimate of the general factor saturation of a test and allows the evaluation of the extent to which composite scale scores are interpretable as a measure of a single common factor (Reise, 2012). To test item convergent and discriminant validity, we employed multi-trait scaling analysis with the two criteria: (1) convergent validity is supported when an item-domain correlation is greater or equal to 0.40 or; (2) discriminant validity is supported when item-domain correlation is higher than that with other domains (Hays and Hayashi, 1990).

## Results

To establish the dimensionality of the instrument, we conducted an exploratory factor analysis model under the item response theory paradigm. We tested a unidimensional model against a two-, three-, and four-dimensional models. **Table 1** displays four information criteria and log-likelihood parameters for the fitted models. The comparisons between the nested models using a deviance test showed that the difference between the models up to model 3 was significant. In addition, the AIC, the AICc, the BIC, and the SABIC decreased when moving to the three-dimensional model and increased when moving to the four-dimensional model. Therefore, analyses indicated a three-factor solution. The variance explained by the three factors for the unrotated solution was 67.40%. The variance explained by each factor of the three-factor solution was, respectively, 41.80, 17.80, and 7.80%. **Table 2** displays the rotated factor loadings. Absolute factor loadings greater than 0.40 were considered salient. The three dimensions were labeled: Membership, Emotional connection, and Opportunities.

**Table 1:** Results from unconditional maximum likelihood factor analysis under the item response theory.

						Comparing models			
Model	AIC	AICc	SABIC	BIC	Log*L*	Models	Δχ^2^	Δ*df*	*p*
1	22047.25	22053.57	22126.19	22284.98	-10973.62				
2	21383.57	21392.44	21476.72	21664.09	-10632.78	1 versus 2	681.682	9	<0.001
3	21099.08	21110.61	21204.86	21417.63	-10482.54	2 versus 3	300.491	8	<0.001
4	21149.25	21163.43	21266.09	21501.10	-10500.63	3 versus 4	-36.178	7	>0.05

*LogL, log-likelihood; AIC, Akaike information criterion; AICc, corrected AIC; BIC, Bayesian information criterion; SABIC, Sample-Size Adjusted BIC. The number of the model corresponds to the number of dimensions.*

**Table 2:** Rotated factor loadings.

	Opportunities	Emotional connection	Membership
Item 1	0.173	0.015	**0.756**
Item 2	-0.067	0.001	**1.001**
Item 3	0.192	-0.132	**0.537**
Item 4	-0.048	**-0.798**	0.041
Item 5	0.014	**-0.979**	-0.059
Item 6	0.069	**-0.565**	0.223
Item 7	**0.896**	0.005	-0.025
Item 8	**0.826**	0.021	0.009
Item 9	**0.664**	-0.044	0.059
Item 10	**0.620**	-0.028	0.052

*Coefficients greater than ∣0.40∣ are in bold face and retained for that factor.*

The full-information three-factor confirmatory IRT model exhibited good fit indices, M_2_^∗^(2) = 2.321, *p* = 0.313, RMSEA = 0.013 (95% CI: 0.000–0.075), CFI = 0.99, TLI = 0.99. Using confirmatory factor analysis, we compared the fit of three models: a unidimensional model, a three-dimensional correlated factors model, and a bifactor model. **Table 3** displays the results which indicate that the three-dimensional correlated factors model provides a significant improvement in fit relative to the unidimensional model. In addition, the log-likelihood test and four information criteria suggest that the bifactor was an improvement over the three-dimensional correlated factors model. Therefore, by any information index as well as the log-likelihood test, the bifactor model exhibited the largest improvement in fit when compared to the other two models.

**Table 3:** Comparison of multidimensional item response theory models using confirmatory factor analyses.

						Comparing models			
Model	AIC	AICc	SABIC	BIC	Log*L*	Models	Δχ^2^	Δ*df*	*p*
1	23765.54	23771.39	23848.07	24006.86	-11832.77				
2	22935.38	22941.97	23022.86	23191.19	-11414.69	1 versus 2	836.158	3	<0.001
3	22877.36	22885.87	22976.40	23166.96	-11378.68	2 versus 3	72.016	7	<0.001

*LogL, log-likelihood; AIC, Akaike information criterion; AICc, corrected AIC; BIC, Bayesian information criterion; SABIC, Sample-Size Adjusted BIC. The number of the model corresponds to: (1) a unidimensional model, (2) a three-dimensional correlated factors model, and (3) a bifactor model.*

**Table 4** shows results of DIF analyses. None of these results was statistically significant. The only notable exception was the first item concerning age group-based DIF.

**Table 4:** Results of DIF analyses.

	Gender			Age group		
	*W*	*df*	*p^∗^*	*W*	*df*	*p^∗^*
Item 1	2.614	1	0.353	8.934	1	0.028
Item 2	0.019	1	0.891	0.156	1	0.866
Item 3	1.258	1	0.374	3.135	1	0.255
Item 4	0.186	1	0.788	1.835	1	0.351
Item 5	3.546	1	0.331	1.837	1	0.351
Item 6	3.373	1	0.331	0.279	1	0.853
Item 7	1.452	1	0.374	0.000	1	0.999
Item 8	0.139	1	0.788	0.006	1	0.999
Item 9	1.538	1	0.374	1.074	1	0.500
Item 10	1.286	1	0.374	3.726	1	0.255

*^∗^Benjamini–Hochberg adjustment.*

DTF was evaluated by examining the differences in expected total scores for gender as well and age group differences given the same latent trait levels. In terms of gender differences, the sDTF value was 0.44 (95% CI = -15.52 to 20.26) and the uDTF value was 1.64 (95% CI = 1.20-20.38). The sDTF was not statistically significant overall (*p* = 0.968). Concerning age group-based differences, the sDTF value was 2.26 (95% CI = -16.55 to 18.05) and the uDTF value was 2.55 (95% CI = 1.39–18.58). Here too, the sDTF was not statistically significant overall (*p* = 0.818).

**Table 5** reports descriptive statistics, reliability, and intercorrelations among study variables. Reliability was satisfactory for all study variables. In particular, Cronbach’s alpha was equal or higher than 0.80 for the three subscales and the total scale of our measure of sense of community in the school. In addition, we calculated omega total (ω*_t_*) and omega hierarchical (ω*_h_*). Omega total for total score was 0.92, while omega total for subscales Opportunities, Emotional connection, and Membership was, respectively, 0.82, 0.83, and 0.86. Omega hierarchical was 0.71. The SoC scale and its three subscales were significantly related to well-being. The effect size of the correlations coefficients was generally medium according to Cohen (1988). These findings provided evidence of criterion-related validity to a reasonable degree. Statistically significant but negligible point-biserial correlation coefficients were observed between gender (female) and the scale and subscales of sense of community at school. The point-biserial correlation coefficients between age group and the scale and subscales of sense of community at school were negative and statistically significant, meaning that student attending middle school reported higher scores compared to students attending high school. The effect size of the correlation coefficients was generally medium according to Cohen (1988).

**Table 5:** Descriptive statistics, reliability, and intercorrelations among study variables.

	*M*	*SD*	α	1	2	3	4	5	6	7	8	9
1. Gender^a^	–	–	–	–								
2. Age group^b^	–	–	–	–	–							
3. Membership	3.22	0.89	0.82	0.08^∗∗^	-0.19^∗∗^	–						
4. Emotional connection	3.41	0.92	0.80	-0.02	-0.25^∗∗^	0.44^∗∗^	–					
5. Opportunities	2.93	0.86	0.80	0.06^∗^	-0.38^∗∗^	0.62^∗∗^	0.36^∗∗^	–				
6. SoC-S	3.16	0.71	0.86	0.05^∗^	-0.35^∗∗^	0.84^∗∗^	0.72^∗∗^	0.85^∗∗^	–			
7. Class satisfaction	3.59	0.79	0.73	-0.05^∗^	-0.15^∗∗^	0.47^∗∗^	0.54^∗∗^	0.39^∗∗^	0.57^∗∗^	–		
8. Relationships with teachers	3.12	0.93	0.81	0.04	-0.29^∗∗^	0.55^∗∗^	0.28^∗∗^	0.55^∗∗^	0.58^∗∗^	0.35^∗∗^	–	
9. Well-being	3.28	0.68	0.86	-0.06^∗^	-0.17^∗∗^	0.48^∗∗^	0.41^∗∗^	0.41^∗∗^	0.54^∗∗^	0.48^∗∗^	0.45^∗∗^	–

*^∗^p > 0.05; ^∗∗^p > 0.01. ^a^Gender was coded 1 for males, 2 for females; ^b^age group was coded 1 for middle school, 2 for high school. SoC-S, Scale of Sense of Community in the School. The Mental Health Continuum–Short Form (Keyes, 2006; Keyes et al., 2008; Lamers et al., 2011) as the measure of well-being.*

**FIGURE 1:**
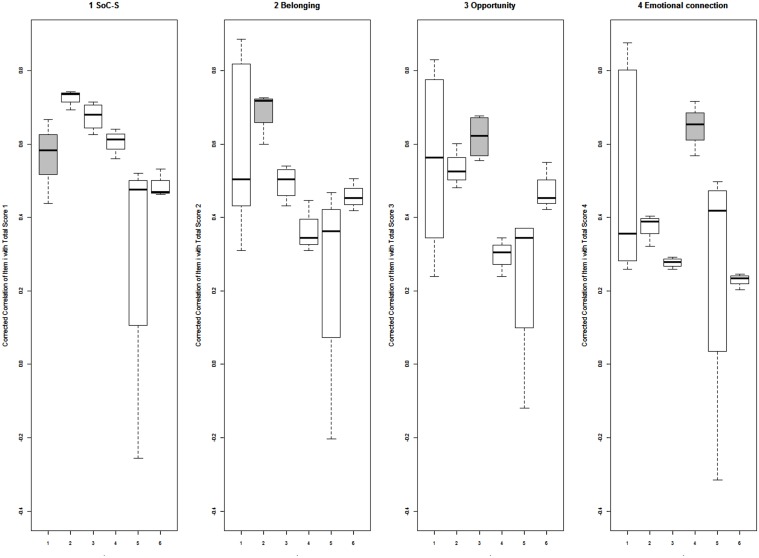
Box plots of corrected item-total correlations. Correlation coefficients appear top-to-bottom if on the *y*-axis, while the scales and the subscales left-to-right if on the *x*-axis: (1) Scale of Sense of Community in the School, (2) Membership, (3) Opportunities, (4) Emotional connection, (5) Class satisfaction, and (6) Relationships with teachers.

To investigate item convergent and discriminant validity, we employed multi-trait scaling analysis including the total score of SoC-S, the three subscales, Class satisfaction, and Relationships with teachers. **Table 6** displays corrected item-total correlations. All the item-domain correlations were greater than 0.40. In addition, all the item-total correlations were higher with their own total scale or subscale than with the other subscales or scales. Items belonging to the same total scale or subscale correlated highly amongst themselves. **Figure 1** summarizes these results. Specifically, the gray boxes of the box plots (corresponding to the corrected correlation of items with their own scale or subscale) are above (i.e., correlation coefficients are higher) the white boxes (corresponding to correlations of items with the other scales or subscales). Moreover, since CFA is a valuable analytic tool for construct validation, we performed a full-information five-factor confirmatory IRT model including the following five scales: Membership, Emotional connection, Integration and fulfillment of needs, Class satisfaction, and Relationships with teachers. The fit of the model was satisfactory, M_2_^∗^(58) = 248.205, *p* < 0.001, RMSEA = 0.04 (95% CI: 0.04–0.05), CFI = 0.95, thus, providing compelling evidence of the convergent and discriminant validity of theoretical constructs. Taken together, these findings provided support for convergent and discriminant validity.

**Table 6:** Corrected item-total correlations.

	SoC-S	Membership	Opportunities	Emotional connection	Class satisfaction	Relationships with teachers
Item 1	0.67	0.88	0.60	0.32	0.37	0.52
Item 2	0.65	0.89	0.53	0.39	0.37	0.45
Item 3	0.61	0.82	0.48	0.40	0.42	0.42
Item 4	0.44	0.31	0.24	0.86	0.34	0.17
Item 5	0.51	0.34	0.30	0.88	0.38	0.22
Item 6	0.54	0.45	0.34	0.80	0.44	0.29
Item 7	0.63	0.54	0.83	0.29	0.34	0.42
Item 8	0.61	0.52	0.82	0.28	0.35	0.43
Item 9	0.56	0.49	0.77	0.28	0.32	0.54
Item 10	0.52	0.43	0.76	0.26	0.32	0.39

*SoC-S, Scale of Sense of Community in the School.*

## Discussion

The importance of sense of community in the school has been recognized in the literature (e.g., Pretty et al., 1994, 1996; Battistich et al., 1995; Battistich and Hom, 1997; Baker, 1998; Osterman, 2000; Bateman, 2002; Vieno et al., 2005, 2007, 2013). However, there is a need for a theory driven reliable and valid assessment instrument. To fill this gap, the purpose of this study was to examine the psychometric properties of a scale of sense of community in the school that was based on McMillan and Chavis’ (1986) theoretical formulation and its adaptation to the population of adolescents (Chiessi et al., 2010). Specifically, the current study has three main aims: (a) to investigate factorial structure and reliability of a scale of sense of community in the school; (b) to assess DIF and DTF of the scale; and (c) to examine discriminant, convergent, and criterion validity of the scale.

As expected, exploratory and confirmatory factor analysis revealed three dimensions that were labeled: Membership, Emotional connection, and Opportunities. These three factors are similar to the first three dimensions hypothesized in the model of McMillan and Chavis (1986): membership, emotional connection, and satisfaction of needs.

Concerning the multidimensional structure of the SoC-S, the present study revealed that sense of community in the school can be conceptualized as multidimensional as well as unidimensional. Specifically, a bifactor model provided the best fit to the data and the results support the presence of a general factor as well as the relatively distinct nature of the three dimensions. As for scoring, the bifactor model supports the use of scores based on both the primary dimension and the sub-subscales. Therefore, researchers and practitioners can focus on either the total score or the scores on Membership, Emotional connection, and Opportunities.

As far as differential item/scale functioning is concerned, the findings of the present study revealed that girls and boys as well students attending middle school and those attending high school responded in largely similar ways to the measure. In other words, participants with the same standing on the latent variable assessed by SoC-S, but sampled from these different groups (i.e., groups based on gender or age group), do have equal expected scores on this scale. There was only one exception: the first item of the scale did exhibit DIF by age group. However, we can conclude that there is no substantial bias in the scale. Thus, SoC-S can be used to compare scores between these different groups. If differences based on gender or age group are substantiated in future studies, such evidence would indicate that these differences are most likely due to socio-psychological processes associated with gender and educational level rather than to the psychometric properties of the instrument (e.g., gender socialization processes affecting the nature of the social relationships that are established by female and male adolescents). In the present study, and contrary to what has been reported for residential sense of community (Chiessi et al., 2010), we have found that female students reported slightly higher scores on SoC-S than their male counterparts, suggesting that the school context may facilitate close relationships with specific peers that are more typically searched for by females. Moreover, the levels of SoC-S were higher among middle school students than high school students. These results are consistent with those on residential sense of community, which decreases from first to late adolescence (Cicognani et al., 2006; Albanesi et al., 2007; Chiessi et al., 2010). Future studies should investigate the possible reasons behind these differences.

In terms of the third aim of the present study, the SoC-S demonstrated good discriminant, convergent, and criterion validity. Specifically, in line with previous research on the relationship between sense of community referred to the residential community and well-being (Davidson and Cotter, 1991; Pretty et al., 1996; Prezza and Costantini, 1998; Prezza et al., 2001; Chipuer et al., 2003; Farrell et al., 2004; Albanesi et al., 2007; Vieno et al., 2007; Prati et al., 2015), SoC-S was associated with well-being. In addition, SoC-S showed evidence of discriminant validity in its relationship to the theoretically different, but related constructs of satisfaction with the classroom environment and students’ relationships with their teachers (Cicognani and Albanesi, 2015). Finally, Cronbach’s alpha, omega total, and omega hierarchical revealed that SoC-S has good reliability.

Some caution is necessary when interpreting the findings of the present study because probability sampling methods were not used to recruit participants. However, the present study makes use of a relatively large sample of students attending many schools potentially representative of the Italian social background. Another limitation of our research is that our study sample included one Italian-speaking country. Therefore, additional research is needed to assess the validity of the measure in other countries and languages. Moreover, the fact that intercorrelations among the subscales (ranging from 0.36 to 0.62) were comparable to the correlations with the discriminant measures (ranging from 0.28 to 0.55) may suggest relatively low discriminant validity, given that the scales are supposed to measure relatively different constructs. However, this conclusion was not supported by the findings of Multitrait-Multimethod approach of scale validation. This approach is more robust and reliable than those obtained from the correlation in the investigation of convergent and discriminant analysis. The results of CFA provided additional evidence of the convergent and discriminant validity of theoretical constructs. Another limitation of the present study is that we did not use a convergent measure. Two considerations guided our choice. First, the items of the SOC-S were adapted from an instrument assessing sense of community on adolescents with established psychometric properties. Second, to our knowledge, there are not validated instrument aimed at assessing sense of community in the school.

Despite these limitations, we believe that the development of the SoC-S has many potential implications for future research. First, researchers and practitioners can rely on a scale of sense of community in the school whose psychometric properties have been examined and are satisfactory. Second, the development of items for the SoC-S was guided by theoretical considerations. As we discussed in the Section “Introduction,” many studies on sense of community in the school literature made use of *ad hoc* measures. Third, SoC-S was specifically developed for the study of sense of community in the school and allows for the use of a composite score as well as subscale scores for Membership, Emotional connection, and Opportunities. Fourth, the use of the same instrument could help researchers and practitioners to understand and compare the findings across different studies. Fifth, SoC-S provide a measure to evaluate interventions at the school level, unlike classroom sense of community scales which are more appropriate for the evaluation of interventions at the class level. For instance, SoC-S may be particularly relevant to assess the impact of programs aimed at preventing phenomena such as bullying, since this behavior tends to occur in relatively unsupervised areas such as the playground (e.g., Olweus, 1993; Craig and Pepler, 1998). In conclusion, the SoC-S has good psychometric properties and the findings suggest that the instrument has potential in both research and practice. This measure with sound psychometric properties will be a valuable tool in future research and intervention on a range of important outcomes for students, such as delinquent behavior or bullying behaviors.

## Author Contributions

GP contributed to conception or design of the work. EC and CA collected data. All authors involved in data analysis and interpretation. GP drafted the article. EC and CA critically reviewed the article. All authors approved the final version of the manuscript to be published.

## Conflict of Interest Statement

The authors declare that the research was conducted in the absence of any commercial or financial relationships that could be construed as a potential conflict of interest.
