# Supplementary material for: Psychometric Properties of a Multidimensional Scale of Sense of Community in the School
Source: Front Psychol. 2017 Aug 29;8:1466. doi: 10.3389/fpsyg.2017.01466 (PMC5581826; doi:10.3389/fpsyg.2017.01466)
Supplement: Supplementary file 1 [file Data_Sheet_1.pdf]

Appendix

The Scale of Sense of Community in the School (SoC-S)

Think about your school.

Below you can find some statements that we want you to consider in relation to your school.

Please read them carefully and indicate with a cross (X) how TRUE each statement is for you.

Warning! Remember that the numbers have the following meaning:

1 = not at all true

2 = not very true

3 = quite true

4 = very true

5 = completely true

1. I think that this is a good school (Credo che questa sia una buona scuola)
2. I am proud to belong to this school (Mi sento fiero/a di far parte di questa scuola)
3. I feel safe in my school (In questa scuola mi sento sicuro/a)
4. I spend a lot of time with other students attending this school (Passo parecchio tempo con gli altri ragazzi/e che frequentano questa scuola)
5. I like to stay with other students attending this school (Mi piace stare con gli altri ragazzi/e della mia scuola)
6. In this school, I feel I can share experiences and interests with other students (In questa scuola, sento di poter condividere le mie esperienze e i miei interessi con gli altri ragazzi/e)

7. In this school, there are enough initiatives for me (In questa scuola ci sono abbastanza iniziative)
8. In this school, there are many initiatives which can involve the students (In questa Scuola ci sono parecchie iniziative che coinvolgono gli studenti)
9. My school provides adequate counseling and support services for students (La scuola offre adeguati servizi di sostegno e aiuto agli studenti)
10. Students are involved in organizing a variety of school events (Gli studenti sono coinvolti nell'organizzare eventi scolastici)
